# Laser-Accelerated Proton Beams as Diagnostics for Cultural Heritage

**DOI:** 10.1038/srep40415

**Published:** 2017-03-07

**Authors:** M. Barberio, S. Veltri, M. Scisciò, P. Antici

**Affiliations:** 1 INRS-EMT, 1650 Boul. Lionel Boulet, Varennes, Canada; 2 Secondary Sources Divisions, ELI-ALPS, Tsiza Lajos krt, 85-87, Szeged, Hungary; 3 INFN / University of Rome ≪La Sapienza≫ P.zzale A. Moro 5, 00161 Rome, Italy

## Abstract

This paper introduces the first use of laser-generated proton beams as diagnostic for materials of interest in the domain of Cultural Heritage. Using laser-accelerated protons, as generated by interaction of a high-power short-pulse laser with a solid target, we can produce proton-induced X-ray emission spectroscopies (PIXE). By correctly tuning the proton flux on the sample, we are able to perform the PIXE in a single shot without provoking more damage to the sample than conventional methodologies. We verify this by experimentally irradiating materials of interest in the Cultural Heritage with laser-accelerated protons and measuring the PIXE emission. The morphological and chemical analysis of the sample before and after irradiation are compared in order to assess the damage provoked to the artifact. Montecarlo simulations confirm that the temperature in the sample stays safely below the melting point. Compared to conventional diagnostic methodologies, laser-driven PIXE has the advantage of being potentially quicker and more efficient.

In the last few decades, a large effort has been put into applying innovative Physics and Chemistry research techniques for diagnostic and conservations of objects of interest for Cultural Heritage. There are many groups worldwide which are currently exploring the possibility of developing equipment for the diagnostic and conservation of artifacts^1^,^2^ where the main challenge is to obtain the most information available without causing damage^3^. Classical diagnostic, conservation, restoration and consolidation techniques typically require removal and transportation of the artwork from a museum or an archeological site to a laboratory or micro-sampling of the artwork for analysis^4^. Chemical information on artworks (ceramic, bronzes, metals, pigments) is obtained using surface spectroscopies including Photoluminescence, Raman, X-ray photoelectron spectroscopy (XPS), X-Ray-Fluorescence (XRF), Energy Dispersive X-ray Fluorescence (EDX) in SEM. Morphological information can be obtained using Scanning Electron Microscope (SEM)^5^. The complete chemistry of the bulk material is retrieved using more sophisticated (and expensive) nuclear physics techniques such as Proton Induced X-ray and Gamma Emission (PIXE and PIGE)^6^,^7^.

In the classical PIXE and PIGE, heavy charged particles (i.e. protons, alfa-particles or sometimes heavy ions) are used to create inner-shell vacancies in the atoms of the specimen. As in the X-ray fluorescence spectroscopy and electron probe microanalysis, the X and Gamma-rays, produced by de-excitation of the vacancies, can be measured by an energy-dispersive detection system which gives a characteristic fingerprint of each chemical element that is present in the analysed bulk sample. The incident charged-particle beam, typically consisting of protons with a mean energy of 1–5 MeV, is classically produced by a small Van de Graaff accelerator or a compact cyclotron. The advantage of using PIXE (in the following we will mention only PIXE, but the same applies for PIGE when considering Gamma-rays) with respect to other X-ray spectroscopies is that protons, compared to X-rays, can be focused and guided by electrostatic or electromagnetic devices/optics and thus can be transported over large distances without provoking any loss in the beam intensity (pencil scanning). As a result, the incident fluences on the samples are generally much higher in the PIXE than in ordinary, true-excited XRF (X-ray Fluorescence). Moreover, PIXE allows preforming an analysis with variable spatial resolution, since protons can be focused down to a beam diameter in the micrometer range. Moreover, the relative detection limits of PIXE are typically two orders of magnitude better than in XRF and other electron spectroscopies (EDX or Auger). Currently, PIXE is used for the analysis of a wide range of materials from proteins to cells and tissues, from polymers to ancient pigments and artefacts. Typically, in the classical PIXE analysis of protein or tissues, an incident proton beam (mean energy ~2.5 MeV and beam current ranging from 10 nA to 150 nA) generates a spectrum with an X-ray count rate in the order of 800–2000 counts/seconds^8^.

However, all these diagnostics have several limitations. Raman and Photoluminescence spectroscopy techniques require sophisticated spectrometers and lasers^9^. SEM and XPS must be performed under vacuum conditions. PIXE and PIGE require conventional particle accelerators (with beam energies typically ranging from a few keV to maximum a few MeV) that are located in dedicated laboratories, since their operation requires particular analysis conditions (e.g. ultra-high vacuum conditions and strongly controlled temperature)^10^. However, all these techniques allow only the study of the first superficial layers of the material bulk and therefore limit the analysis to the corrosive surface patina or to the decoration, without giving important information of the material inside. Additionally, they are able to analyze merely small surfaces (beam spot sizes are generally in the order of tens of μm^2^). This makes a complete analysis on a larger surface very time consuming (using a pencil-scanning analysis).

PIXE and PIGE spectroscopy performed in typical facilities devoted to Cultural Heritage studies, such as with the AGLAE^5^ facility located at the French Louvre Laboratory C2RMF^11^ or INFN-LABEC laboratory^12^,^13^ located in Florence with a conventional proton accelerator (energies 1 to 5 MeV, beam current from 10 pA to nA), are using spot sizes ~10 μm (up to 500 μm). This results in numerous point measurements (10–100) with each point taking around 100–9000 s to compile the complete information. The deriving long analysis time can produce proton damage to the artifacts.^14^ The maximum analysis depth that can be reached on those facilities is between 2–20 microns. This depth is typical of biological film, surface patina, or bronze cancer (cuprite ad malachite). The analysis is therefore limited to deteriorated layers without obtaining information on the pristine material bulk. Moreover, these conventional diagnostic methods are not easily tunable and adaptable (typically, tuning the energy of the accelerated beam takes at least tens of minutes) which often reduces their application to only a certain field of energy range and to micrometric surface areas.

Scanning a larger bulk volume allows identifying more quickly if there are chemical elements that require further investigation (e.g. harmful elements). If needed, a more precise investigation can then be performed with higher resolution, i.e. a smaller spotsize and a more precise depth, in the considered volume (e.g. in order to find harmful elements). On the contrary, if no harmful elements could be detected on the larger volume, this means that the scanned area is “clean/healthy”, and the analysis can continue on another part of the artefact.

The advent of high-power, ultra-short lasers has opened up possibilities of laser-based particle acceleration, including protons^15^,^16^. The investigation and application of these laser-accelerated beams is currently challenging many research laboratories worldwide, in particular for the improved characteristics of these sources such as compactness, efficiency, versatility and tunability. Laser-based particle beams present the advantage of having a high current (kA), strong laminarity at the source (emittance about 100 times better than conventional accelerators), short duration (ps scale), and small source size (tens of μm)^17^. Current multi-hundred-TW table-top laser systems can generate on-target intensities of ~10^19^–10^20^ W/cm^2^ with resulting proton energies of ~15–20 MeV with a typical laser-to-proton energy conversion efficiency of 1–6%^18^,^19^,^20^ and a current in the kA regime (each laser shot produces up to 10^13^ protons). These parameters make them a desired candidate for innovative applications requiring one or more of the above-mentioned properties, including medicine^21^,^22^, fusion^23^, radiography^24^ or injectors in larger scale accelerators^25^,^26^.

In this paper we show that laser-accelerated proton beams can be used as diagnostic in the field of Cultural Heritage and their use can have considerable advantages over conventional PIXE and PIGE spectroscopies. In particular, laser-based protons have the advantage of allowing (1) a complete chemical analysis on a larger volume of the artworks (analyzed surface in the order of cm^2^); (2) a deeper and more precise “layer by layer” analysis, obtainable by tuning the beam energy from few MeV to tens of MeV within a very short timescale; (3) and, depending on the facility, a higher punctual dose (obtained in one or more shots).

Here we concentrate on improving diagnostic techniques that are performed with non-portable, quite costly facilities (accelerators), since in this case the criteria of portability for both technologies (conventional and laser) is not applicable and as such a direct comparison is justified (current table-top lasers obtaining laser-driven MeV protons are still not yet portable, but conventional accelerators in this energy range neither). Our setup is similar to a typical PIXE setup used in the field of Cultural Heritage, with the only difference that the conventional ion source is replaced with a laser-based one (see Fig. 1, detailed setup is described later): The high-power laser is impinging a commercially available solid target which acts as proton source. The laser-generated protons are used to irradiate a sample positioned at a variable distance from the source. The X/Gamma-rays, produced in the interaction between the laser-generated protons and the sample to be probed, are detected by a X/Gamma-ray detector that is monitoring the interaction region. Our approach and experimental methodology has a strong technical similarity with other recently developed techniques aiming at using laser-generated protons in different applications, particularly medical applications. Different pioneering papers have tackled issues that are linked to our approach and experimental methodology, such as: (1) availability in air (obtained e.g. using a 7.5 μm-thick polyimide vacuum window^27^ or 23 to 50 μm Mylar or Kapton window^28^,^29^,^30^; (2) suppression of parasitic radiation (obtained e.g. using a deflecting magnet in order to avoid unwanted X-ray radiation from the target interfering with the direct source-aperture line-of-sight^27^,^28^, or stopping potential contributions from low-energy carbon ions using a thick foil)^30^; (3) fast energy selection (obtained e.g. using chicane of magnetic dipoles^27^,^28^ or a combination of Radio-Frequency Cavity and quadrupole-sequence^31^). In this work we have not emphasized these adjustments since they have already been covered by the above-cited references.

In order to assess the heating effect of the impinging protons to the samples, we have performed some preliminary simulations. The interaction between the different laser-generated proton spectra and the sample target was simulated with a two-dimensional Monte Carlo code, in which we inserted as proton heating source a laser-generated proton beam obtained in previous published experiments (see references later): The laser-generated proton beam was modelled as the projection of a proton virtual point source with diverging rays, generating a proton source with diameter of 50 μm. This modelling of a proton source is similar to what described in ref. 32 with the laminarity of the beam calculated as indicated in ref. 33. The divergence half-angle divergence of the proton beam (α) has been adjusted depending on the considered proton energy, as obtained in ref. 34. Within the opening angle, all particles were uniformly distributed. This modelling is standard in the field of laser-plasma interaction for measuring proton-induced heating effects^35^.

Since the laser-driven proton yield is heavily dependent on the laser-facility, we need to consider different laser scenarios which - for simplicity – we rationalize as being able to be represented by the following four different laser-facilities used for laser-driven proton production: (1) Very high-energy, longer pulse laser – currently difficult to obtain commercially such as the LLNL-TITAN laser (maximum energy: up to 220 J, typical pulse duration: 700 fs, central wavelength: 1.056 μm, repetition-rate ≪ 1 Hz)^36^,^37^; (2) high-energy laser, long pulse laser – currently difficult to obtain commercially, but not out of reach for industry, such as the LULI-ELFIE (30 J, 350 fs, 1.056 μm, rep-rate ≪ 1 Hz)^38^; (3) High energy, short pulse laser – similar to what can be obtained commercially as 1 PW laser (e.g. from Amplitude Technologies or Thales Optoelectronics), such as the ASTRA-GEMINI (10 J, 45 fs, 800 nm, envisioned rep-rate for future facilities 5–10 Hz (e.g. at the Extreme Light Infrastructure)^39^); and finally (4) High-energy, short pulse laser – commercially available as 100–500 TW laser (e.g. from Amplitude technologies) such as the FZD-DRACO laser (5 J, 25 fs, 800 nm, rep-rate 10 Hz)^40^. A comparison of the different spectra obtained from the different laser-facilities (and extracted from the cited papers) is shown in Fig. 1B. The TITAN spectrum has been obtained during the experimental campaign described later.

As a starting point for our simulations we use the spectra having the most particles, i.e. those obtained with the TITAN laser spectrum, since having a high proton flux should allow performing a “one-shot” PIXE analysis. On the other side, it is likely that such a particle flux will be heating the irradiated target to the highest temperatures (“worst case scenario”). We focus firstly on the main material categories of interest in the Cultural Heritage, which include bronzes, marbles (stone carbonates), noble metals (gold, silver) and ceramics. In the simulations we position the sample at different distances from the proton source and evaluate the heating effect. We need to ensure that the temperature within the sample stays safely below the melting point. On the other side, the more proton flux irradiates the sample, the more X-ray emission will occur which will improve the signal-to-noise ratio of the PIXE diagnostic. In our setup conditions and for the ceramic sample (which is one of the most critical items with melting point ~1600 °C), this distance is found to be about 6 cm from the proton source (see Fig. 2A). Temperature maps obtained for respectively a marble, silver and ceramic sample (as reference for the most common CH materials), 50 ps after the irradiation using the TITAN proton spectrum, are shown in Fig. 2B. One can see that within the entire sample the temperature stays safely (~25%) below the melting point for all the considered materials (the melting points for marble, silver and ceramic are respectively about 1400, 960 and 1600 °C). We check the temperature map after 50 ps, since the heating onset time for the sample is in the order of 10–30 ps^41^ and, as such, after about 50 ps the sample has reached its maximum temperature during the heating process. Simulations also reveal that the temperature stays almost constant for about 1 ns before the cooling phase starts and the sample cools down almost completely in a few tens of ns.

We validate the simulations obtained for the above mentioned case by a series of experiments performed on the TITAN laser of the Jupiter Laser facility (Laurence Livermore National Laboratory - LLNL), producing laser pulses of about 220 J in 700 fs and operating at a wavelength of 1.054 μm^36^. The laser beam, focused down to about 9 μm focal spot diameter (FWHM) - producing an on target intensity of I~10^20^ W/cm, was used for interacting with a commercially available solid target in order to accelerate protons in the laser-forward direction using the TNSA^42^ mechanism. The Amplified Spontaneous Emission (ASE) has been measured to be < 10^−6^ in contrast, i.e. delivering ~10 mJ in energy. As proton source targets, we used commercially available solid 15 μm Au or Al targets manufactured by Goodfellow, depending on the artefact to be probed (for all samples unless the Au sample, an Au foil was used). The incident laser was tilted by 10° with respect to the target normal direction in order to differentiate between the trajectories of the protons stemming out normally from the back surface, and the electrons following the laser-direction. As proton diagnostics, we used two calibrated Thomson parabolas (TPs) and spectrometers located at 0° (TP 0°) and 9° (TP 9°) with respect to the main pulse laser axis to measure the forward generated proton spectrum. The TPs were placed respectively at a distance of 690 and 565 mm from the proton source (distance to the entrance slit). Proton spectra measured by the TPs were readout in an absolute manner^43^,^44^ using Image Plates (IP) of the type BAS-TR 2025 from Fuji Photo Film Co. Ltd that were analyzed using a FUJIFILM FLA-7000 reader. Additional measurements of the proton spectra were obtained using Radio Chromic Films (RCFs) of the type HS that allowed obtaining a beam spatial distribution. A typical laser-generated proton spectrum obtained during the experiment is shown in Fig. 1B. The laser-generated protons were impinging the sample with an incidence angle of 10°.

As samples we used pure silver (97%, thickness 200 μm, impurities of Cr, Ti and Cu, manufacturer: Goodfellow), gold and bronze (manufacturer Goodfellow, thickness 200 µm: gold 99.95%, bronze Pb < 200, Sn 4.5–7.5%, P 200–4000, total impurities 2000, Cu balance), pure Carrara marble (provider: Le pietre srl), and one ancient ceramic (taken from the medieval archaeological situ of Nicastro, South of Italy, see Fig. 3A; provider: Sovraintendenza ai Beni Culturali of the Region of Calabria, Italy). The latter is a decorated fragment of an amphora (Fig. 3A) dated year 1650 AD^45^. While metals do not alter over time, this is not the case for typical ceramics. All the samples were located at 6 cm from the proton source. At first, we verified the effect of the proton irradiation on the target surfaces for the above-described setup. This has been performed by morphological and chemical analysis on all the irradiated materials before and after the irradiation. All tests performed on the irradiated samples positioned in the above-described setup indicate nonperturbative morphological or chemical changes on the artefacts. Figure 3 shows, as example for all, the results obtained for the ceramic sample, which is the most critical sample we have irradiated. Morphological analysis, conducted by optical microscope indicate the absence of fractures and cracks on the surface, while chemical analysis, conducted by EDX analysis under SEM conditions, show the absence of chemical changes on the materials surface and inside the bulk (within an analysed depth of 10 µm). Verifying the XRF emission before and after irradiation (see Fig. 3B) shows a slight decreasing of the Ca, Fe and Cu lines. However, this effect is not recognizable by eye and occurs also in conventional irradiation facility conditions. This said, similar to when applying conventional methods, it is no longer possible to obtain the dating of the artefact, because the high-cumulated dose during the PIXE strongly affects the thermoluminescence.

As specified in ref. 46, the thermoluminescence (TL) methods for analysing most artefacts are based on the amount of radiative dose cumulated during the artefact’s lifecycle (Paleodose). In detail, the sample age is evaluated as the ratio between the Paleodose (specified in Gy) and the Dose Year (Gy/year) of the archaeological site. The irradiation by high energetic protons can significantly change the radiation cumulated into the finding, changing the Paleodose and affecting the dating process. We checked the (non) applicability of thermoluminescence dating techniques using the conventional method as described in ref. 45. Figure 3C shows the TL emission before (dark line – 100x magnified) and after laser-driven proton irradiation (blue line). Our artefact would now be dated with an age of 140000 years instead of 324. However, also conventional PIXE methods invalid the use of dating mechanisms, hence there is no difference in this diagnostic when using laser-driven or conventional PIXE. Having assessed the suitability of the distance between the sample and the proton source (6 cm) for not damaging the artifact, we have tested the photon emission stimulated by the laser-accelerated proton beam. The PIXE produced by the laser-generated protons was measured at an angle of 10° with an X-ray spectrometer sensitive to photons with energies ranging from few keV to 50 keV^46^ and using as detection medium IPs of the type BAS-TR2025 which were readout by the same IP scanner as for the proton diagnostic (see before). Behind the artefacts we placed a stack of RCFs (of the type HD) in order to verify the centering of the proton beam onto the artefact. Due to the limited sensibility range of the spectrometer, we tested the validity of our method only on the silver sample, since all other materials have their K-emission energies outside the main detectable spectral range of the spectrometer. The results of a single-shot PIXE conducted on silver are indicated in Fig. 4. As visible from the RCF positioned behind the sample (see Fig. 4A), the silver target was fully covered by the proton beam, i.e. the protons irradiate a surface of several cm^2^. The IP of the PIXE spectrometer (see inset in Fig. 4, Fig. 4B) shows three well-defined bands corresponding to the Ag K (first order) and L (second order) bands, as evaluated from the Bragg law:


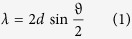



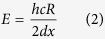


where, λ and E are the wavelength and the energy of the X-rays, Θ the diffraction angle, x the distance of the line from the zero-order, *d* is the lattice parameter of the crystal (in our case 0.68 nm), R is the distance of the IP-crystal (in our case 0.1 m). The convoluted spectrum (Gaussian convolution, obtained using the cross sections for each line)^47^ is shown in Fig. 4C. The leftmost line, close to the zero-order (the intense round spot in Fig. 4B), corresponds to the first order K-alpha line (22.2 keV) while the two adjacent lines on the right side can be attributed K-alpha lines of Ti and Cr respectively (4.93 and 5.94 keV). The *one-shot* PIXE data are in perfect agreement with those obtained by conventional XRF on the same sample (Inset in Fig. 4C), which indicate the presence of the L-alpha line of Silver (3 keV) and K-alpha lines of Ti (4.93 keV), Cr (5.94 keV), and Cu (8.97 KeV). The above-obtained results confirm the feasibility of laser-driven PIXE, which can be performed on a larger surface than what can be achieved conventionally, in addition to being obtained in a single sub-ns laser shot.

We have performed the experimental validation using laser-generated protons obtained on the TITAN laser, which is probably not the most suitable laser for performing routinely this kind of analysis. More appropriate lasers for ensuring a broader use of this application are those that are commercially available and have a higher repetition rate^39^,^40^. On those facilities, the integrated number of protons is typically at least one order of magnitude lower than what was obtained on the TITAN laser (see Fig. 1B). However, the lack of proton flux can easily be balanced by cumulating a certain number of shots, and with this reproducing the same X-ray signal as obtained during one single shot on the TITAN laser. A proton flux deficit ratio of 100 on a 10 Hz laser can be balanced with 100 consecutive shots, ideally lasting 10 s in a high-repetition rate target setup. Given the lower proton flux, the temperature conditions (i.e. that the samples stays safely below melting point) are still warranted. Even when using higher-repetition rate lasers (e.g. up to 100 Hz), the time interval between two consecutive shots is sufficiently long to ensure that the temperature within the sample cools down and the cumulative effect of the shots does not provoke any damages to the artifact even at higher repetition rate operation.

A last point to consider is the more punctual analysis on different layers (depths) within the material. This analysis is needed if the sample exhibits zones that require further (more precise) investigation (e.g. harmful elements). A potential way to perform this is using an energy selector, i.e., a device that only selects a fraction of the proton beam within the entire broad-band spectrum around a tunable central energy and reduces the energy-spread of the particles^48^,^49^. Since protons deposit most of their energy at the end of their trajectory (Bragg peak), this device allows performing the analysis on different layers with a depth precision depending on the allowable energy spread. An additional advantage is that this kind of “laser-plasma” energy selector can easily and rapidly change its central energy (the main element for the energy selection being a movable slit, which selects the central energy out of the broad-band proton spectrum and can potentially be moved with sub-s velocity) and thus allows a quicker scan than what obtained using conventional accelerators where each central energy adjustment requires a full reassessment of the accelerator. Regarding an analysis of the artifact using a smaller spotsize, this can be obtained e.g. by putting irises in front of the artifact or using focusing techniques^50^.

Since the technique strongly relies on the detection of peaks within the X-ray emitted spectrum (see Fig. 4B and C), it is not easy to make a general statement about the speed and efficiency of laser-driven PIXE. The three key parameters for performing the technique are (1) number of emitted photons per shot (which depend on the impinging proton quantity), (2) acceptance of the X-ray diagnostic, (3) sensitivity of the detector. In our case a single TITAN-laser shot and a spectrometer with good acceptance (solid angle of about up to 1e-3) was sufficient to identify a clear signal on a very sensitive Imaging Plate. On other, lower energy (but higher repetition) lasers, it might be that a few shots would be needed in order to have such a clear signal – evidently depending on the sensibility of the detector and acceptance of the spectrometer. Technology will make necessary advancements in these fields, once this technique will be more routinely available.

We have not considered in the present study other materials of interest for Cultural Heritage such as glasses (and silicates in general), wood, paper, and one of the most commonly used pigments, the “Realgar” (Arsenic Sulfide As_4_S_4_). Testing those materials with the current setup (ultra-high-vacuum conditions) was difficult, since to perform PIXE of those materials the best approach is testing them in air and this requires a more sophisticated setup, which we did not have at disposal. This will be part of future studies.

## Additional Information

**How to cite this article**: Barberio, M. *et al*. Laser-Accelerated Proton Beams as Diagnostics for Cultural Heritage. *Sci. Rep.* **7**, 40415; doi: 10.1038/srep40415 (2017).

**Publisher's note:** Springer Nature remains neutral with regard to jurisdictional claims in published maps and institutional affiliations.

## Figures and Tables

**Figure 1:**
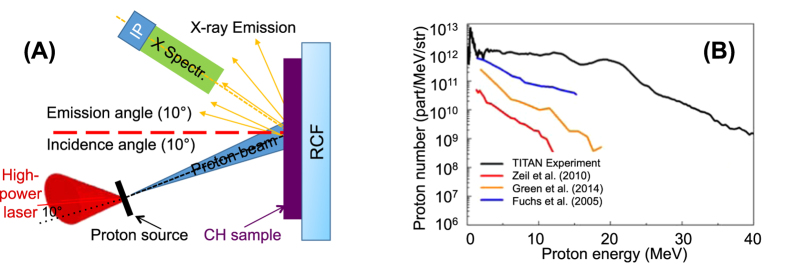
(**A**) Experimental Setup used for one-shot PIXE; (**B**) Comparison of different proton spectra obtained for 4 different laser-facilities operating in different energy/pulse duration ranges. The TITAN spectrum was obtained during the experimental campaign; all other spectra are extracted from the cited works in the text.

**Figure 2:**
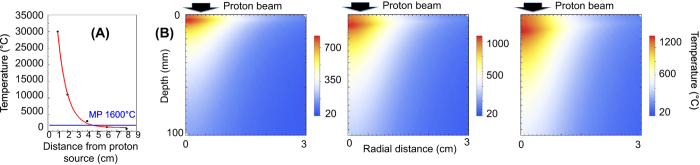
(**A**) Simulated maximum temperature vs. distance from the proton source for a ceramic artifact when irradiated with the laser-generated proton beam as obtained on the TITAN laser (MB indicates the melting point of 1600 °C). (**B**) Temperature maps, obtained with the Monte Carlo code, of a silver, marble, and ceramic sample irradiated by the laser-generated proton beam as obtained on the TITAN laser. The images show the temperature 50 ps after irradiation. The target is located at 6 cm from the proton source. The 0 level in the figure indicates the target surface in front of the proton beam, with the protons impinging from the top. Only the first 100 μm of the target surface are shown.

**Figure 3:**
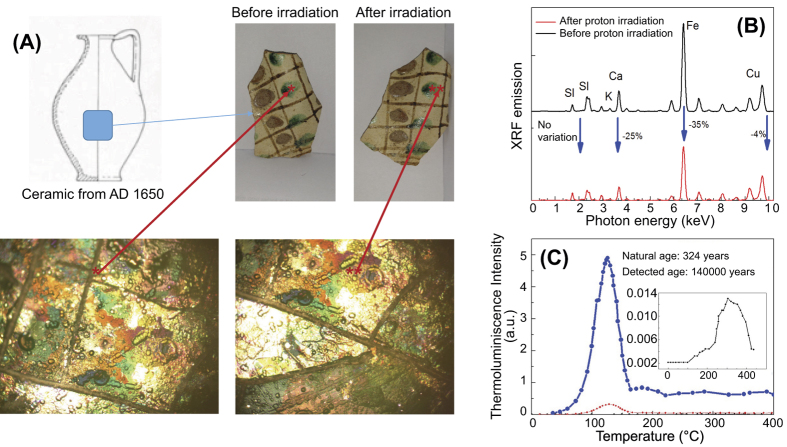
(**A**) Archeological situ and details about the ceramics used for testing the damaging effect of the laser-generated protons during the one-shot PIXE; Images show the artifact before and after the irradiation; (**B**) XRF optical image of the sample before and after proton irradiation; (**C**) Results of the dating process performed before and after irradiation.

**Figure 4:**
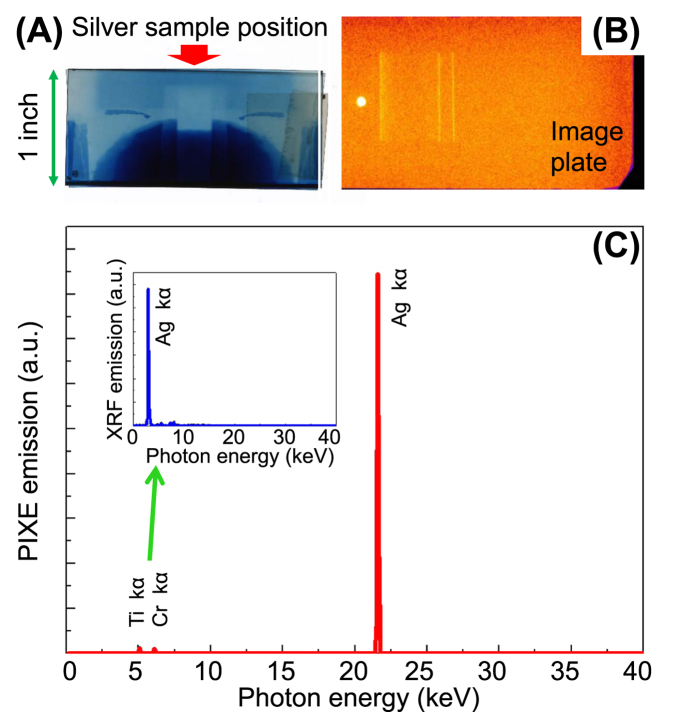
(**A**) Silver sample mounted in front of the RCF indicating the footprint of the laser-generated proton beam; (**B**) ImagePlate (IP) positioned behind the X-ray spectrometer showing the different lines and the zero-order; (**C**) Convoluted PIXE spectrum obtained by Bragg analysis on the IP shown in (**B**). The inlet of (**C**) shows the XRF analysis.
